# Learning Microbial Community Structures with Supervised and Unsupervised Non-negative Matrix Factorization

**DOI:** 10.1186/s40168-017-0323-1

**Published:** 2017-08-31

**Authors:** Yun Cai, Hong Gu, Toby Kenney

**Affiliations:** Department of Mathematics and Statistics, Dalhousie, Halifax, Canada

**Keywords:** Microbial communities, Subcommunities, Metagenomics, Non-negative Matrix factorization

## Abstract

**Background:**

Learning the structure of microbial communities is critical in understanding the different community structures and functions of microbes in distinct individuals. We view microbial communities as consisting of many subcommunities which are formed by certain groups of microbes functionally dependent on each other. The focus of this paper is on methods for extracting the subcommunities from the data, in particular Non-Negative Matrix Factorization (NMF). Our methods can be applied to both OTU data and functional metagenomic data. We apply the existing unsupervised NMF method and also develop a new supervised NMF method for extracting interpretable information from classification problems.

**Results:**

The relevance of the subcommunities identified by NMF is demonstrated by their excellent performance for classification. Through three data examples, we demonstrate how to interpret the features identified by NMF to draw meaningful biological conclusions and discover hitherto unidentified patterns in the data.

Comparing whole metagenomes of various mammals, (Muegge et al., Science 332:970–974, 2011), the biosynthesis of macrolides pathway is found in hindgut-fermenting herbivores, but not carnivores. This is consistent with results in veterinary science that macrolides should not be given to non-ruminant herbivores. For time series microbiome data from various body sites (Caporaso et al., Genome Biol 12:50, 2011), a shift in the microbial communities is identified for one individual. The shift occurs at around the same time in the tongue and gut microbiomes, indicating that the shift is a genuine biological trait, rather than an artefact of the method. For whole metagenome data from IBD patients and healthy controls (Qin et al., Nature 464:59–65, 2010), we identify differences in a number of pathways (some known, others new).

**Conclusions:**

NMF is a powerful tool for identifying the key features of microbial communities. These identified features can not only be used to perform difficult classification problems with a high degree of accuracy, they are also very interpretable and can lead to important biological insights into the structure of the communities. In addition, NMF is a dimension-reduction method (similar to PCA) in that it reduces the extremely complex microbial data into a low-dimensional representation, allowing a number of analyses to be performed more easily—for example, searching for temporal patterns in the microbiome. When we are interested in the differences between the structures of two groups of communities, supervised NMF provides a better way to do this, while retaining all the advantages of NMF—e.g. interpretability and a simple biological intuition.

## Background

Microbes affect human physiology and global nutrient cycling, through the action of microbial communities [1–3]. A microbial community usually consists of hundreds or even thousands of different microorganisms [4, 5] which survive through the interaction with each other and environments and form metabolically integrated communities [6]. Although in some cases the abundance of a single species can have a big effect on the overall state of the community, for example some species of pathogens are believed to single-handedly cause illnesses, in many cases, differences between different types of microbial communities (for example, the communities in the guts of healthy and IBD people) are attributable to the overall structure of the community. It is therefore critical to devise models which take into account this overall structure.

Next generation sequencing has generated a large amount of microbial metagenomics data for the study of microbial diversity of different environments. These data consist of either marker-gene data (counts of OTUs) or functional metagenomic data, i.e. counts of reaction-coding enzymes. The OTUs or gene counts will be referred to as variables, and a sample will be also referred to as an observation in this paper. Considering the difficulty of collecting data and the large number of variables, the data always consist of hundreds or even thousands of variables but only a few observations, which means *p*≫*n* (*p* is the number of variables and *n* is the number of observations). In addition, many species are only observed in a few samples; thus, the data are highly sparse [7, 8]. This makes it challenging to apply classical statistical analysis methods.

Exploratory data analysis, such as principal component analysis (PCA) [9], on the original data matrix, is not appropriate for count data and has largely been replaced by clustering analysis or principal coordinates analysis based on UniFrac [10]. The UniFrac distance measures the abundance difference between two samples, incorporating phylogenetic tree information between the organisms. Although UniFrac is widely used, it has some drawbacks. One is that it does not address the heterogeneity between samples due to the different sequencing depths for different samples. Subsampling techniques are sometimes used to attempt to remedy this problem, but these do not fully resolve the problem and involve throwing away a large amount of information in the data and so are not recommended [11]. UniFrac based methods are only applicable to OTU data, not whole metagenome sequence data. Furthermore, UniFrac is an ad hoc method in that it is not based on a probablistic model and thus does not provide as much insight as an explicit statistical model-based approach.

Early work on the probabilistic modeling of microbial metagenomics data by [12] has represented the data as multinomial samples from an underlying multinomial distribution which in turn is generated from one of several Dirichlet mixture components. The hyperparameters of each of the Dirichlet mixture components have been assumed to follow a Gamma prior. This Bayesian probability framework seemed to be reasonable, though some assumptions such as choice of prior are arbitrary; however, the analysis results of the two examples based on this probability framework in [12] are not totally convincing in that the clustering results of lean and obese samples do not really show clustering patterns, and the method underperforms existing methods at classification. Another Bayesian probabilistic framework models the contaminated sample as a mixture of several known microbial community sources [13]. Bayesian Inference of Microbial Communities (BioMiCo) [14] is a more recent Bayesian hierarchical mixed-membership model. BioMiCo takes OTU abundances as input and models each sample as a two-level hierarchy of mixtures of multinomial distributions which are constrained by Dirichlet priors. This model identifies clusters of OTUs which it calls assemblages and then infers the mixing of assemblages within samples. Unlike the Gamma prior used in [12], the Dirichlet priors are used to control the sparsity of mixing probabilities for both levels of the multinomial distributions which results in more interpretable assemblages and a more parsimonious model.

The above probability frameworks have been mainly applied to the marker-gene data, but could easily be applied to whole metagenomic data as well. Another hierarchical Bayesian framework, BiomeNet [15], has been developed to specifically model the structure of metagenomic data.

A common theme in these Bayesian probability modelling frameworks is that each sample is modeled as a mixture of several typical “types”. These typical types are mostly inferred from data by computational methods. The Bayesian framework provides a natural vehicle for fitting complicated models, but the resulting models are generally not easy to interpret because of the hierarchical structure, and the computation usually takes a very long time.

In order to provide an effective exploratory data analysis method that is suitable for both marker-gene and functional metagenomic data and is based on a probability model that can capture the subcommunity structure information and can address the issues of heterogeneity among samples, we explore the application of Non-negative Matrix Factorization (NMF) to microbiome data in a likelihood framework. NMF has been widely applied in many areas, such as image and natural language processing, and also has found many applications in computational biology [16]. More recently, it was applied in the ocean microbes data to investigate the biogeography of microbial function and its correlation to environmental distance [17]. It has also been applied to metabolic profile matrices [18]. This application is similar to the unsupervised NMF we used here. They focused on functional gene reads aggregated into pathways in that paper, rather than direct reads or OTU data. It also seems that they used NMF on the proportion data, rather than the original counts. This is theoretically not correct, as using the original counts allows the estimate to account for the fact that samples with greater sequencing depth give a more accurate estimate of the proportions. Conceptually, similar to the above Bayesian modeling frameworks, NMF models each sample as a mixture of different types. These types represent the structure of subcommunities. Instead of using a multi-level hierarchical structure as in BioMiCo [14] and BiomeNet [15], NMF uses one level of subcommunities as building blocks which makes the connection between the sample microbiome composition and the OTUs or reaction-coding enzymes more direct; this will provide better interpretability for the analysis results. In addition, NMF is a natural method to use for dimension reduction and feature selection in microbiome data. The commonly used unsupervised learning methods such as PCA and vector quantization (VQ) for reducing dimension and picking up the main features of the data usually result in linear combinations with negative coefficients which are hard to interpret naturally in this context. We want to find the main features (subcommunities) of the data and at the same time keep all the elements in these features non-negative. The features extracted by NMF are somewhat different from those identified by PCA or other variable selection methods; they are points in the high dimensional space which form a convex hull to envelop the observed points. Thus, they can involve much more than a single variable or a few variables. As demonstrated by Lee and Seung [19], NMF also tends to identify sparse features, and thus, each sample is expressed as a non-negative linear combination of a few sparse points (types), which further facilitates the interpretation of the results.

Like PCA or BiomeNet, NMF is an unsupervised method. Although NMF can extract the main features from the data, it cannot guarantee that these features are the best discriminant features to distinguish different classes. For example, if two classes are described by similar features, NMF will extract an average of these features to fit both classes, rather than separate features for the two classes.

For the purpose of identifying differences between different types of communities, we develop a supervised version of NMF in this paper. In cases where a single variable (or a small number of variables) is the main discriminant feature, this is often readily apparent from the types identified. In other cases, where the main differences are based on smaller-scale community-wide structure differences, NMF is able to identify these. In the real-data examples, we study some examples where the main differences between the classes come from a small number of key variables, and other examples where the main differences seem to arise more in the structure of the whole community. In these latter cases, the features extracted by NMF represent subcommunities of microbes that act as building blocks for the whole community.

There are many off-the-shelf supervised learning methods that can perform a classification directly on such data (a review is given by [8]). Since typically *p*≫*n* (the number of predictor variables is substantially larger than the number of data points), we need to choose methods designed for the *p*≫*n* case. Directly applying these classification methods often results in quite good classification. With some classification methods (for example random forest and the elastic net), variable selection is also possible. However, the selected variables are often difficult to map back to some discriminating community level features between classes, particularly if the true discriminating feature is not a single variable. Some classification methods (such as support vector machine, boosted trees or Neural network) can construct a very good classifier for such data, but without any possibility of interpretation and thus cannot provide any insight for the underlying community structure. BioMiCo [14] builds a classifier on the discriminant assemblages of the OTUs to predict the class labels with these assemblages showing the subcommunity structures. The model complexity of BioMiCo is controlled by the number of assemblages and the Dirichlet priors which are both pre-specified. These pre-specified parameters in principle can be adapted to the data through cross-validation on the training data, but running these Bayesian models needs a long time for each run which hurts the wide applicability of BioMiCo to different data.

Since we are interested in the community level features or systematic differences between different classes, we first use NMF to identify features from each class and then we build a classifier based on the weights distribution of each sample on the combined features from different classes. The features selected by this method will describe the original data well and also contain classification information. We can measure how well the features identified relate to the differences between different types of communities by looking at the prediction error of classifiers. As mentioned above, the purpose of NMF is to provide insights into the structural differences between different types of microbial communities, rather than to produce the most accurate classification possible. Classification is however a good measure to gauge the extent to which the subcommunities identified have important biological roles in the overall community structure.

Supervised NMF has similar model structures to BioMiCo but is fast to compute, and the only tuning parameters are the number of features that are extracted from different classes. Unlike BioMiCo which controls the sparsity of variables within features by the Dirichlet priors, the sparsity of NMF is decided by the number of features. With fewer features used in the model, each feature tends to be less sparse and conversely more features mean each feature is more sparse.

## Materials and methods

We will first give a review of NMF and its application to metagenomic data under the Poisson likelihood framework. We then describe the idea of supervised NMF based on unsupervised NMF, with the computation of the weight matrix over the combined features, followed by the method used to choose the tuning parameters for the supervised NMF. The details of the prediction method are given in the next subsection.

### The NMF model

Non-negative matrix factorization [19] is a dimension reduction method for non-negative data. The idea is to represent each data point as a linear combination of non-negative features which are also computed from the data. Given a non-negative *p*×*n* matrix *X*, we approximate *X* by *TW*, where *T* is a non-negative *p*×*k* matrix, referred to as the *type* (feature) matrix, and *W* is a non-negative *k*×*n* weight matrix. Each column of *X* is approximated by a non-negative linear combination of the types (columns of *T*). Here, *k* is the number of types or features which determines the complexity of the model; thus, it is a tuning parameter in this context. Usually, *k* is chosen such that (*p*+*n*)*k*≪*np*, so that we reduce the dimension significantly.

In our analysis, *X* is the microbes data with counts of OTUs or genes. Specifically, *X*_*ij*_ is the number of times the *ith* OTU or gene is observed in the *jth* sample. Thus, each feature (column) in *T* describes a subcommunity and each column in *W* contains the linear coefficients for the corresponding sample (column) in *X*. The whole community in a sample is thus approximated by a mixture of the subcommunities. For count data, such as our *X*, we model each element as an independent Poisson observation given its mean in the matrix *TW*. Note that because the Poisson mean varies between samples, the proportions of each OTU will exhibit the sort of overdispersion commonly seen. The idea is that there is a latent proportion of OTUs given as a weighted mean of the types, but the observation is a Poisson sample with this mean. We might argue that the sequencing procedure actually introduces more variance, so introducing overdispersion to the measurement distribution may have some value in future work. The covariance structure between the variables in *X* is implicitly given by the patterns in the type matrix *T*. The columns of the type matrix *T* are constrained to have the sum 1, and in this context, each column in *T* can be interpreted as the composition of OTUs or genes for each *type*. The different sequencing depths for the samples in *X* are absorbed in the weight matrix *W*. To compute *T* and *W*, we maximize the Poisson log-likelihood of the data [7],

$$L(T,W)=\sum_{i,j}\left(X_{ij}\log(TW)_{ij}-(TW)_{ij}\right), $$

In most literature (e.g. [20]) Euclidean distance is used as a criterion, assuming a Gaussian distribution for the observations instead of the Poisson.

There are a number of algorithms available for fitting NMF, for example [19, 21–24]. A thorough discussion of the algorithms available and their merits can be found in [25]. We used the R package NMF by Renaud [26], which implements the algorithm of Lee and Seung [19]. The choice of algorithm can, in theory, influence the results because the solution to NMF is not always unique, since the criterion depends only on the product TW. Usually, in practice, the non-negativity constraint will ensure that there is a unique solution.

Another challenge in applying NMF is to choose, *k*, the number of types. Generally, the log-likelihood increases with *k* increasing. We can plot the log-likelihood values versus *k* to find the “elbow point” after which the log-likelihood increases more slowly. This means the increase in the number of types will not add as much in modeling the data. Thus, we should choose the *k* value at the elbow point. In cases where there is no such elbow point, exploring multiple different *k* values by using our interactive data exploration tool, *SimplePlot* which is described later in this section, could help to find the *k* value based on which some meaningful data structure can be shown.

### Supervised NMF

For a supervised learning problem, we have observations from different classes. Our objective is usually to find the differences between the structures from the different classes. We will approach this by separately identifying the subcommunities in each class first and then combine them into a single matrix of subcommunities. Each sample now can be expressed as a mixture of all these subcommunities. For example, if data *X* has *g* classes,

$$X=\left(X^{(1)},X^{(2)},\cdots,X^{(g)}\right) $$

where *X* ^(1)^,*X* ^(2)^,⋯,*X* ^(*g*)^ are *g* classes of observations. From *X* ^(*i*)^, we can calculate the non-negative type matrix *T* ^(*i*)^ and weight matrix *W* ^(*i*)^ (*i*=1,⋯,*g*) by NMF. To get the hidden structure of different classes in the whole data, we combine these type matrices together and denote this combined type matrix for the whole data as

$$T=\left(T^{(1)},T^{(2)},\cdots,T^{(g)}\right) $$

It is straightforward that *T* is non-negative since each *T* ^(*i*)^ is non-negative. For fixed *T*, to maximize the Poisson log-likelihood for the whole data *X* is equivalent to maximizing the Poisson log-likelihood for each sample, because the weight vectors in *W* associated with different samples are independent. Thus, calculating the weight matrix *W* can be reduced to performing a non-negative Poisson regression of each sample in *X* on *T*. The details of the procedure are given in Appendix 1.

### Method for choosing the number of types

The number of types for each class of observations should be chosen to best describe its own class but not to describe other classes or noise. For discrimination purposes, the number of types for each class should be chosen to best separate the classes in combination with the number(s) of types in other classes. The most direct way to choose number of types for all classes is to find the model mis-classification errors on the validation sets for each combination of the numbers of types for different classes. However, the computation burden is heavy in such an effort. Thus, we propose to choose the number of types for each class separately first and try the selected combinations of number of types for different classes if the results are not clear-cut. Full details of the method used to choose the number of types, with some explanation are presented in Appendix 2. The basic method is to fit an NMF model on training folds from one class and compare the deviances on the test fold from that class with the deviances on a fold from each other class, using a Wilcoxon Rank-Sum test, then combining the test statistics for each fold into a single test statistic and estimating the standard deviation from the results for different folds.

In easy cases, the number of types to choose is clear-cut. Often, the number of types will be clear-cut for some classes, but not others. In these cases, we fix the number of types for the easy class(es) and use cross-validated error to choose the number of types for the other class(es).

### Prediction

For fixed *T*=(*T* ^(1)^,*T* ^(2)^,⋯,*T* ^(*g*)^), we apply the non-negative Poisson regression algorithm on training data to calculate the training *W* and on test data to calculate the test *W*. After getting the *W* matrix, we have effectively reduced the dimension from *p* to *k*, in the sense that for the fixed *T* feature matrix, each observation is best approximated by the corresponding *k* vector in the *W* matrix. We can use an off-the-shelf supervised learning method to predict the class labels since *k*<*n*. Note that the sum of each column in the *W* matrix is the same as the sum of corresponding column in the *X* matrix, which means sequencing depth in microbiome data. When we perform a supervised learning, the transpose of the *W* matrix will be used as input for each observation. Geometrically this corresponds to projecting all the data into the space spanned by the vectors in the *T* matrix. The entries for different individuals on the same input vector of *T* are not comparable due to the different sequencing depth for the original data. We normalize the *W* matrix so that its column sum is 1 before performing a supervised learning method. This makes the entries in each row of *W* comparable and also makes it possible to show all the data in a plot. The normalization at this step is different from the normalization on the *X* matrix directly, because different sequencing depths result in heterogeneity in the original observations, and this has to be taken care of in the likelihood calculation and in the estimation of *T* and *W*.

We choose a suitable supervised learning method based on the graphical display of NMF results as described below. In the following sections, we most often perform a logistic regression on *W*. We choose logistic regression because our interactive exploration of the data suggests that a linear classifier is appropriate for this classification, and logistic regression is one of the simplest linear classification methods. The trained logistic regression model can then be used to do prediction on the test *W*.

### Graphical display of NMF results

To properly display the NMF results we need to project down to two dimensions. A software package, *SimplePlot*, has been developed by one of the authors in this paper. It is available from Toby Kenney’s website www.mathstat.dal.ca/~tkenney/SimplePlot/. Using *SimplePlot*, we can interactively choose a projection. Since the projection of the *W*-matrix is entirely determined by the projections of the types, the program allows us to manually move the positions of the types (represented by crosses on the figure) around the plane and watch how the relative positions of vectors from the *W*-matrix change. The advantage of using interactive software is that it is easier to identify non-linear separation if that is more appropriate for a particular dataset.

## Results and discussion

We apply both unsupervised NMF and supervised NMF on three datasets: whole metagenome sequences from faecal samples from 39 mammals (the mammal dataset) [27]; time sequences of 16S data from a range of body sites across two individuals (the moving picture dataset) [4]; and whole metagenome sequences from IBD patients (some Crohn’s disease, some ulcerative colitis) and healthy controls (the Qin dataset) [28]. We gain some biological insight through the biological interpretation of the features and graphical display of the weight matrices from the NMF analysis. NMF is compared with UniFrac [10] and supervised NMF with two commonly used classification methods: Support Vector Machine (SVM) and Random Forest (RF). For the SVM, linear kernel, polynomial kernel and radial basis kernel are used. We use the R package e1071 [29] to apply SVM and the R package randomForest [30] to apply RF. The two tuning parameters for SVM—gamma and cost—are chosen by minimizing the average cross-validation error as the best combination for four values from 10^−4^ to 0.1 for the gamma and three values from 1 to 3 for the cost. We also compare the moving picture dataset results with BioMiCo [14] and the Qin dataset results with BiomeNet [15].

### The mammal data

The mammal dataset [27] contains gut metagenomes extracted from *n*=39 mammals. The metagenomes include 1239 different types of genes (categorized by EC number). The mammals can be classified into four types: carnivore, foregut fermenting herbivore, hindgut fermenting herbivore and omnivore. There are 21 herbivores, 11 omnivores and 7 carnivores.

#### Unsupervised NMF results for mammal data

**Fig. 1:**
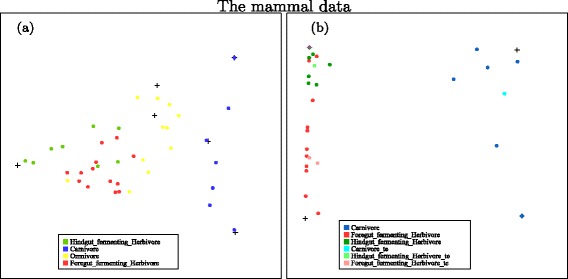
Left: Unsupervised NMF can totally separate the carnivores (blue) from the other three types of animals. The foregut-fermenting herbivores (red), the hindgut-fermenting herbivores (green) and Omnivores (yellow) are largely separated with a few mixed. Right: supervised NMF for separation of the carnivores from the herbivores. Both training carnivores (dark blue) and testing carnivores (light blue) are easily separated from the herbivores. The model was not trained to separate two types of herbivores, but a good degree of separation is shown for the foregut-fermenting herbivores (dark red for training and light red for testing cases) and the hindgut-fermenting herbivores (dark green for training and light green for testing cases). **a** A SimplePlot for unsupervised NMF, **b** A SimplePlot for supervised NMF

We calculate the log-likelihood for a range of *k* values and then observe how the log-likelihood changes with the *k* values. We choose the number of types for the mammal data as nine and apply unsupervised NMF on the data. A snapshot of the projected data on a plane is shown on the left panel of Fig. 1. From the plot, we can see that the carnivores can be totally separated from others and the other three types are mostly separated with a few overlapping points. The dimension of the data is reduced from 1239 to 9 in this analysis.

#### Supervised NMF results for mammal data

We first apply supervised NMF on the whole mammal dataset. The supervised NMF did not improve the classification significantly from the unsupervised NMF in this case. This is possibly because the metagenomic composition of omnivores is a mixture of that of herbivores and carnivores. In order to find the most important discriminant features between herbivores and carnivores, we apply supervised NMF only on the carnivores and herbivores from the mammal dataset [27]. So the data we use here contain metagenomic sequencing of fecal samples from 28 mammals: 7 carnivores and 21 herbivores. As the number of observations is small, we perform a sevenfold cross-validation on the whole data. Each time, we use six folds as training data and the remaining observations as test data.

We find two types that suitably describe both classes. Then, we calculate two types on each class using the training data and combine them to get the type matrix *T*. Fixing the type matrix, we obtain the weight matrices for the training cases and test cases by the non-negative Poisson regression method detailed in the appendix. We fit a logistic regression using the training data weight matrices and perform a prediction on the test data.

The projections of both training and test data in one fold of the sevenfold cross-validation, relative to the positions of four types calculated from the training data, are plotted in the right panel of Fig. 1. It shows that both training carnivores and test carnivores could be well separated from herbivores. Also, from the plot, we can see that although we did not supervise the distinction between the two types of herbivores, there is some reasonable degree of separation between these two classes.

Both the training and test errors are 0 in each fold of the sevenfold cross-validation data split. The prediction errors are all 0 meaning our algorithm could separate the two classes of mammals perfectly. The huge number of variables in the original data could be reduced to four features (two for each class), which means the classes of mammals can be easily determined by four features.

To compare the supervised NMF with support vector machine and random forest, we choose the best tuning parameters for SVM by the same sevenfold cross-validation as in supervised NMF. The best cost value for all kernels is 1. The best gamma value for polynomial kernel is 0.01, for sigmoid kernel 0.001 and for radial basis kernel 0.1. We also compare with Random Forest with the sparse variables removed. (We remove the 50% of the variables with lowest abundance in all samples.) The mean and standard deviation of prediction errors for models with these best tuning parameters on different folds are summarized in Table 1. The table shows that supervised NMF is among the methods which perform perfectly on the mammal data.

**Table 1:** Comparison of test errors for support vector machine with linear kernel (SVM l), with polynomial kernel (SVM p), with sigmoid kernel (SVM s), with radial basis kernel (SVM r), RandomForest (RF), RandomForest with sparse variables removed (RFrm) and Supervised NMF

Dataset	SVM l	SVM p	SVM s	SVM r	RF	RFrm	Supervised NMF
Gut	0	0.2335	0	0.0661	0	0	*0*
Tongue	0.0202	0.2694	0.0202	0.0484	0.0081	0.0242	*0.0040*
Left Palm	0.1245	0.2691	0.1446	0.2691	0.1285	*0.0643*	0.0924
Right Palm	0.3455	0.2724	0.3455	0.1667	0.0732	*0.0488*	0.1789
Mammal	0.0714	0.1428	0.0714	0.1071	0.1429	0.1071	*0*
	[0.0461]	[0.0505]	[0.0461]	[0.0505]	[0.0505]	[0.0505]	[0]
Qin	0.3178	0.3359	0.2592	0.2853	*0.2299*	0.2299	0.2333
	[0.0567]	[0.0530]	[0.0516]	[0.0494]	[0.0573]	[0.0467]	[0.0515]

The first four rows are the prediction errors on the test data. The last two datasets are cross-validated errors with standard errors given in brackets on the line below. Best prediction for each dataset is presented in italics

#### Interpretation of the features in the mammal data

**Fig. 2:**
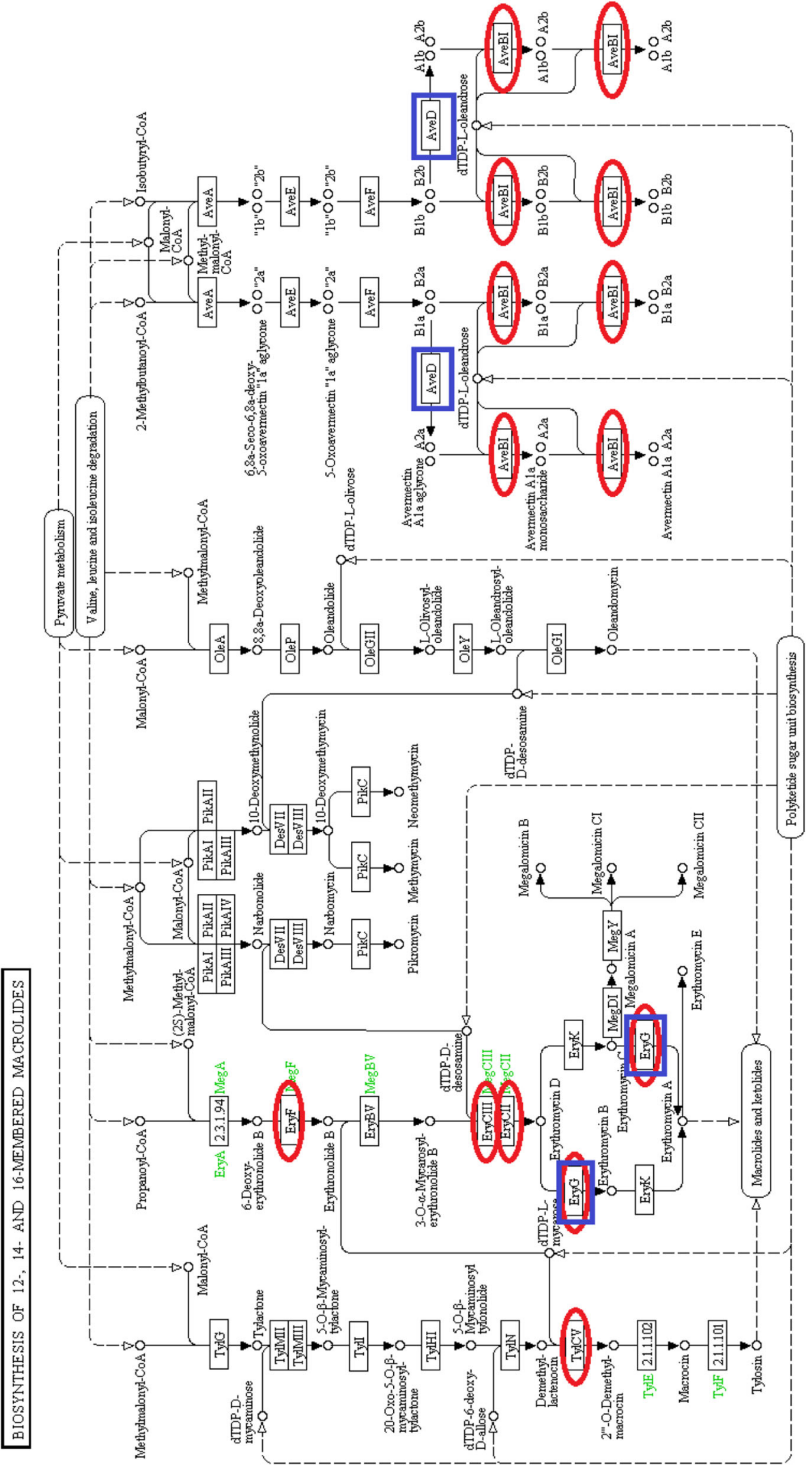
Biosynthesis of 12-, 14- and 16-membered macrolides. Reactions in red ellipses are those appearing in herbivores’ features. Reactions in blue rectangles are those appearing in carnivores’ features. Figure downloaded from KEGG [44], red ellipses and blue rectangles added by the authors

We map the features extracted separately from herbivores and carnivores to the metabolic pathways in KEGG. We find that most of the features from herbivores and carnivores involve the same metabolic pathways except that herbivores have more reactions in the biosynthesis of macrolides pathway, shown in Fig. 2. The most significant difference is found in one of the features of herbivores, which corresponds to the feature (cross) in the upper left corner of the right panel of Fig. 1. (This feature has been highlighted in purple on this plot.) Macrolides are a group of drugs belonging to the polyketide class of natural products. Macrolides are not to be used on non-ruminant herbivores: they rapidly produce a reaction causing fatal digestive disturbance [31]. This explains the results that 8 out of 10 herbivores which have highest weight on this feature are non-ruminants. These correspond to the 8 hindgut-fermenting herbivores (green) in Fig. 1 b. This shows that the inferred differences in the microbial communities of mammals relate well to the known different phenotypes for different mammals.

### The moving picture data

The moving picture data [4] is the most detailed investigation of temporal microbiome variation to date. It consists of a long-term sampling series from two human individuals at four body sites: gut, tongue, right and left palm. Person 2 was measured for a longer time than person 1 (336–373 samples from each body site for Person 2 over a period of 443 days, compared to 131–135 samples from each site for Person 1 over a period of 186 days). The total number of variables (different OTUs) across all samples was more than 15,000. After removing all 0’s, the total number of different OTUs for the gut data is around 3000, for the tongue data is around 2000, for the left palm data and right palm data are around 13,000. In spite of this extensive sampling, no temporal core microbiome was detected, with only a small subset of OTUs reoccurring at the same body site across all time points [4].

#### Unsupervised NMF results for gut data in the moving picture data

**Fig. 3:**
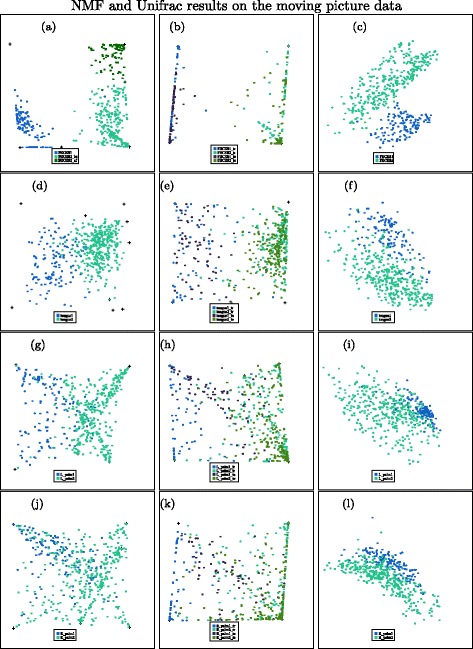
Top row shows results from the gut dataset (with 6 types/coordinates used for the unsupervised methods); second row shows results from the tongue dataset (with 9 types used for the unsupervised methods); third row shows results from the left palm (with 6 types/coordinates); fourth row shows results from the right palm (with 6 types/coordinates). Blue points are from person 1; green points are from person 2. Left: unsupervised NMF; middle: supervised NMF on both training and testing data— darker blue and green points are testing data; right: UniFrac. **a** Gut NMF. **b** Gut supervised NMF. **c** Gut UniFrac. **d** Tongue NMF. **e** Tongue supervised NMF. **f** Tongue UniFrac. **g** L. Palm NMF. **h** L. Palm supervised NMF. **i** L. Palm UniFrac. **j** R. Palm NMF. **k** R. Palm supervised NMF. **l** R. Palm UniFrac

First, we apply NMF to the gut data. The gut data consists of 131 observations from person 1 and 336 observations from person 2. We find the number of types is 6 based on the plot of log-likelihood values versus number of types. And we see that the data from two individuals can be well separated—see the left panel of Fig. 3. It can be seen that the four types seemed to be used to mainly describe individual 2 and two types are mainly related to individual 1. It also shows that the observations for individual 2 are separated into two groups, the reason for which will become clear later in this paper.

#### Supervised NMF results for gut data in the moving picture data

As the gut data is time based, we choose the first 70 time points’ observations out of 131 observations of person 1 and the first 170 time points out of 336 observations of person 2 as training data. If the system changes slowly, we might expect samples from the same individual separated by only a short time might be more closely related. By choosing this separation into training and test data, we minimize the correlation between training and test data, ensuring that we only test the method’s ability to pick up long-term microbial signatures of each individual. A 10-fold cross-validation with the training data split into 10 folds sequentially over time is applied for choosing the number of types and we find two types for each person is the best according to our method. This is an easy classification problem: based on two types for person 1, all deviance values for person 2 are much larger than the deviance values from person 1. A total separation is almost achieved for each fold of the cross-validation for any value of *k* (*k*≥2). This is the same based on two types for person 2. Thus, we choose two types for each person. Then, we fit a logistic regression model on the training *W* matrix and perform a prediction on the test data. The results are shown in the right panel of Fig. 3 and the prediction error is 0 for the test data.

We see that both training and test data are almost perfectly separated between the two individuals which means the distinguishing features of the gut data are included in a matrix consisting of four features. These four features contain sufficient information for classification and will be examined in detail in the interpretation section together with the features computed from the tongue data from these two individuals, because some interesting connections between the tongue data features and gut data features can be detected within individual 2.

#### Unsupervised NMF results for tongue data in the moving picture data

Next, we apply NMF to the tongue data. For the tongue data, there are 135 observations from person 1 and 373 observations from person 2.

It is not obvious what the appropriate value for the number of types should be by looking at the plot of log-likelihood versus number of types. We try NMF on nine types and ten types. Neither achieves good separation between samples from the two individuals. A *SimplePlot* for unsupervised NMF for nine types is shown Fig. 3 d as an example. Here, we see that samples from the two individuals are somewhat separated, but there is a lot of mixing: we cannot achieve a great classification from these features.

Although standard NMF works for the animal dataset and the gut dataset, it does not perform as well on the tongue dataset. The reason is that with unsupervised methods, the signal that is identified is not always the signal we are interested in. Using supervised NMF, we will be able to identify the different features for different classes. This allows us to more easily distinguish samples from different classes.

#### Supervised NMF results for tongue data in the moving picture data

For the tongue data, as above, we choose the first 70 time points’ observations out of 135 observations of person 1 and the first 190 time points out of 373 observations of person 2 as training data. The remaining data are test data. We split the training data over time and perform a 10-fold cross-validation on the training data to find the number of types for both individuals. Our method shows that two types are appropriate for person 1, but is not so clear for person 2 (possibly suggesting nine types). Fixing two types for person 1, and comparing cross-validated error, we choose three types for person 2. This results in a test error of 0.04.

For illustration purposes, we show the *SimplePlot* of both training and test data based on two types for person 1 and three types for person 2 in Fig. 3 f. Through these five features, most of the observations in tongue data could be correctly classified according to which individual they come from.

#### Unsupervised NMF results for left palm data in the moving picture data

For the left palm data, there are 134 observations from person 1 and 365 observations from person 2. We try NMF for several different numbers of types on the left palm data. None of them achieve good separations between samples from the two individuals. A *SimplePlot* for unsupervised NMF for six types is shown in Fig. 3 g as an example. Here, we see that samples from the two individuals are somewhat separated with a considerable amount of mixing.

Standard NMF does not perform well on the left palm dataset. Using supervised NMF allows us to more easily distinguish samples from different classes.

#### Supervised NMF results for left palm data in the moving picture data

For the left palm data, we choose the first 67 time points’ observations out of 134 observations of person 1 and the first 183 time points out of 365 observations of person 2 as training data. The remaining data are test data. Using the same procedure as for the tongue data, we find two types for person 1 and three types for person 2 can best separate the two individuals.

We show the *SimplePlot* of both training and test data based on two types for person 1 and three types for person 2 in Fig. 3 h. Most of the observations in left palm data could be correctly classified with a test error of 0.092.

#### Unsupervised NMF results for right palm data in the moving picture data

For the right palm data, there are 134 observations from person 1 and 359 observations from person 2. Similar to the results for the left palm, there is not a good separation between samples from the two individuals. A *SimplePlot* for unsupervised NMF for six types is shown in Fig. 3 j as an example. Here, we see that most samples from the two individuals cannot be separated using these features.

#### Supervised NMF results for right palm data in the moving picture data

For supervised NMF on right palm data, we choose the first 67 time points from person 1 and the first 180 time points from person 2 as training data. The remaining data are test data. We find two types for each person can best separate the two individuals.

We show the *SimplePlot* of both training and test data based on two types for each person in Fig. 3 k. Most of the observations in the right palm data could be correctly classified according to which individual they come from with a test error of 0.179.

#### Comparisons with other methods

These datasets have also been extensively analysed by BioMiCo [14]. To enable comparison with their results, we reran our analysis with individual months as training data. (Rerunning BioMiCo with our splits into training and test data is infeasible due to its excessive running time.)

We train supervised NMF on different months and predict the identity of the two individuals of all other months. The number of types used for each dataset is the same as we mentioned above. Even though a smaller number of samples are used to train the model, we still get very high-classification accuracy. The accuracy is between 98.1 and 99.8% when using the gut dataset and 85.4 and 92.9% when using the tongue dataset. This is almost the same as BioMiCo’s accuracy, between 98.6 and 99.3% for the gut dataset and between 85 and 93% for the tongue dataset. However, we also get very high accuracy when using the palm data, between 88.9 and 93% when using left palm dataset and between 77.8 and 83% when using right palm dataset. This is significantly higher than BioMiCo’s results (40 to 75%). Palm data are more challenging because human palms are exposed to the external environment. The comparison with BioMiCo concludes that the supervised NMF is not only efficient in terms of computation but also better at finding discriminant features of individuals even with very noisy data.

We also compared supervised NMF with support vector machine, random forest and random forest with sparse variables removed on this moving picture data. We split each body part’s data in the same way as that in supervised NMF. A 10-fold cross-validation is applied to the training part to calculate the best tuning parameters. Models with the best tuning parameters then are trained on the whole training data and used to predict the test data. The results are summarized in Table 1. The comparison for moving picture data shows that supervised NMF gives comparable or better classification results than other methods except for the left and right palm dataset. For these datasets, random forest on the most abundant OTUs performed better than NMF. For the left palm data set, random forest on all variables performed better than NMF.

UniFrac is a widely used unsupervised method. To compare the separation of two individuals, we project the samples on principal coordinates of the unweighted UniFrac distance matrix (based on rarefied samples) in the right-hand column of Fig. 3 with the numbers of the principle coordinates equal to the numbers of types we have used for each case, presented using *SimplePlot*. We can see a clear separation of the two individuals from the gut dataset. Plots of tongue data and palm data show separations to some degree, but not as clear as in our unsupervised NMF plots (left panels in Fig. 3). This shows NMF is an alternative and possibly more useful data exploratory method for such data. In addition, NMF has a natural interpretation in terms of mixtures of communities, but the results from UniFrac are hard to interpret, as they cannot show what causes the grouping effects or where the differences in microbime composition lie.

#### Interpretation of the results

**Fig. 4:**
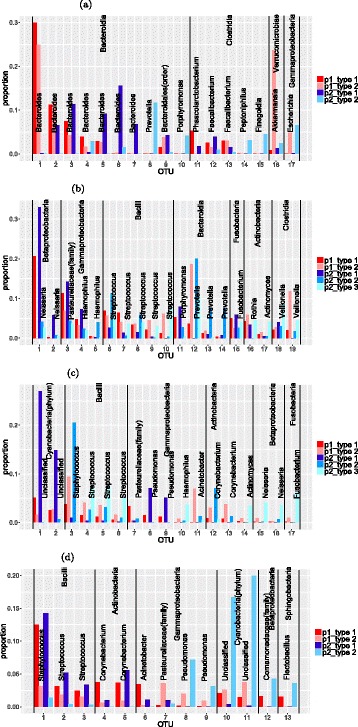
Outstanding OTUs in features of moving picture data: The light and dark red bars are two features from person 1 and the blue bars are features from person 2. The OTUs from the same class are in the same block which is labeled by their class name and the bars are labeled by the genus of the OTUs. The two unlabeled bars in left palm data are the same OTUs with these unlabeled bars in the right palm plots. They are two different unclassified classes in Cyanobacteria phylum. **a** Outstanding OTUs in features of gut data. **b** Outstanding OTUs in features of tongue data. **c** Outstanding OTUs in features of left palm data. **d** Outstanding OTUs in features of right palm data

To examine the main aspects of the features identified, we plot the relative abundance of OTUs for different features in Fig. 4. The feature vectors are of the same dimension as the original observations. A natural side effect of NMF is that the resulting feature vectors are usually sparse. The feature vectors consist of non-negative elements with each vector sum equal to 1. The non-zero values can be interpreted as the percentages of the OTU composition in a particular feature. To get a better illustration, we use a cut-off of 3% for each feature vector in Fig. 4. That is, only those OTUs with above 3% composition in at least one feature are included in the plot.

Figure 4 a shows the main OTUs for the gut data. We find only 17 out of more than 3000 OTUs are larger than the cut-off of 3%. Among these major OTUs, the two features within each individual bear some similarities. But the features between two different individuals are quite different. This is reflected by the fact that several of the most common OTUs in individual 1’s features are not present in individual 2’s features and vice versa. Since each individual’s data can be best represented by his/her own two features and their two features are largely different, this partially explains why the classification of two individuals based on the gut data is an easy problem.

Figure 4 b shows the main OTUs for the tongue data. There are only around 20 OTUs in tongue features above the cut-off of 3%. Again the type matrix of the tongue data is highly sparse. Unlike the features of the gut data, the features of the tongue data for these two individuals are more similar. By looking at the compositions of the most dominant OTUs in each feature, we can easily see similarities between person 1’s type 1 and person 2’s type 1. Also person 1’s type 2 is similar to person 2’s type 2 for OTUs in the classes Fusobacteria and Gammaproteobacteria and similar to person 2’s type 3 for OTUs in the class Bacilli. This suggests that there are similar variation patterns between the two individuals, with the same groups of OTUs increasing or decreasing together. Naturally, the classification for the tongue data is a harder problem.

Figure 4 c shows the main OTUs for the left palm data. Seventeen out of more than twelve thousand OTUs in the left palm features are larger than the cut-off of 3%. Among these major OTUs, the two features within individual 1 have OTUs present and absent together with some variations in their values. The features within individual 2 show a different pattern with each OTU mainly represented by one of the three features. Left palm features within each individual are quite different because the palm’s microbial environment is more variable. Features between individuals are also quite different for most of these major OTUs. Several OTUs in individual 2’s features are not present in individual 1’s features. This may explain why the left palm data can achieve high classification accuracy but lower than the gut data.

Figure 4 d shows the main OTUs for the right palm data. There are only 13 OTUs in right palm features above the cut-off of 3%. The patterns of features within each individual are similar to their left palm data. But features between individuals are more similar except differences in the two unlabeled OTUs. This explains the difficulty in separating two individuals from the right palm data. We also find major OTUs in the right palm features are nearly all present in the left palm features. We do not find the same situation in gut and tongue features. This may be because an individual’s left and right hands are usually exposed to the same environment. It could also be caused by contact between the two hands.

In many of the examples, NMF can act like a variable selection method—identifying individual reactions or OTUs which show different abundances in the two groups of samples. However, in the moving picture tongue dataset, we do not obtain such good classification by looking at individual OTUs. Instead, we look more deeply at the community structure identified by NMF. By examining community-level differences, we were able to classify the individuals with a very high degree of accuracy. We now look in more detail at the communities involved, in an attempt to understand why unsupervised NMF was less effective in this case, and why supervised NMF was able to resolve this problem. This also demonstrates more of the range of interpretability offered by NMF. In addition to highlighting individual OTUs or reactions that differ between the two classes, it is able to isolate bacterial subcommunities from which the microbiome is built up and offer insights into the different structures of these communities.

**Fig. 5:**
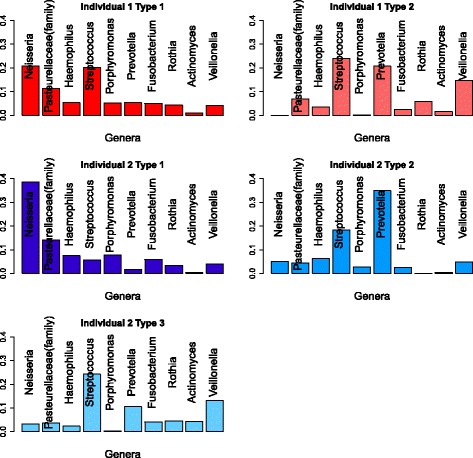
Major genera for tongue feature matrix. The light and dark red bars are two features from individual 1 and the blue bars are features from individual 2. Each bar is labeled by the name of the genus or family

Figure 5 shows the profiles of the types extracted from the two individuals, with graphs of abundance of each genus in that type. For individual 1, we see that type 1 contains higher abundances of *Neisseria*, *Haemophilus*, *Porphyromonas*, *Fusobacterium* and the unclassified genus from the Pasteurellaceae family, while type 2 includes higher abundance of *Streptococcus*, *Prevotella*, *Rothia*, *Actinomyces* and *Veillonella*. This may well be associated with the action of *Porphyromonas*. One species, *Porphyromonas gingivalis*, has been shown in [32] to manipulate the host immune system, allowing pathogens to colonise the community. While the OTU from the genus *Porphyromonas* in this dataset is unclassified at species level, it could have a similar effect to the studied species *P. gingivalis*. This would seem consistent with type 1 having higher levels of various Proteobacteria and Fusobacteria closely related to known pathogens. When we look at the features for individual 2, we see a similar picture, with types representing varying levels of *Porphyromonas*. Again, we see with increased *Porphyromonas*, we have an increase in *Neisseria*, *Haemophilus*, *Fusobacterium*, and the unclassified genus of the Pasteurellaceae family, and a corresponding decrease in *Streptococcus*, *Prevotella*, *Actinobacteria* and *Veillonella*. Type 2 may show that the effect of *Porphyromonas* is non-linear with *Prevotella* actually increasing in abundance with low levels of *Porphyromonas*.

We also examine the types in the absence of *Porphyromonas* (type 2 for individual 1 and type 3 for individual 2). For both individuals, we see that these types are dominated by *Streptococcus*, *Prevotella* and *Veillonella*. However, Fig. 4 b shows the differences between these types. We see that individual 2 has more *Actinomyces* and a different distribution between OTUs within the genus *Veillonella*. Similarly, there are subtle differences between the types with high abundance of *Porphyromonas* (type 1 for both individuals). Individual 1’s type 1 has higher levels of *Streptococcus* than individual 2’s. This might be partially explained by the use of three types to model individual 2, allowing separate types to model both high and low levels of *Streptococcus* in cases with high levels of *Porphyromonas*. However, in Fig. 4 b, we see the presence of higher abundance of a second OTU from the genus *Neisseria* in individual 2’s type 1. This cannot be explained by the different numbers of types used to analyse the two individuals. Supervised NMF is able to identify these subtle differences and use them to identify the individuals, even in situations where the large-scale community structure varies a lot between samples within each individual.

We also consider the idea that the types correspond to communities of microbes. When we look at the type without *Porphyromonas*, we can see the makings of a community structure, with a number of microbes (such as *Prevotella*, *Streptococcus* and *Actinobacteria*) that metabolise glucose into pyruvate, which is later metabolised into lactate, and other microbes such as *Veillonella* which metabolise lactate.

#### Temporal dynamics

**Fig. 6:**
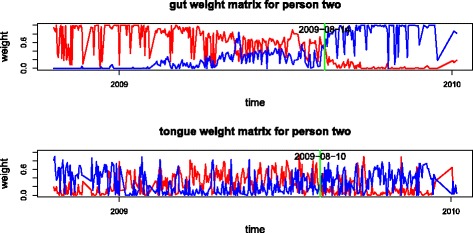
Gut and tongue weight matrix time series plot for person two. The top plot shows the gut weight matrix on the second type (red line) and third type (blue line) from NMF with 3 types. The bottom plot shows the tongue weights on the first type (red line) and the third type (blue line) from NMF for tongue data with 4 types

To investigate the temporal dynamics of the four body sites’ microbiomes, the weight matrices for the gut data and the tongue data are plotted in Fig. 6. When we apply NMF to person 2 with three types for the gut data (see the upper panel of Fig. 6), there is a clear shift at around 2009-08-14. This timepoint is highlighted in Fig. 6. For the gut weight matrix, the dominant weight is initially type 2 and changes to type 3 after this time. For person 2’s tongue data, this shift is not very clear when we use only three types. However, with four types, we can identify a more apparent shift in their weight matrix time series plots. For this data one more feature can bring out more details in the variation of the data. In the lower panel of Fig. 6, the weight matrix time series plots for the tongue data relative to these two features show that type 1 is consistently more represented than type 3 in the early part of the study although not always dominant due to the effects of types 2 and 4; type 3 is more represented than type 1 after the changing point (highlighted on the plot). The shift occurs first in the tongue weight matrix and then can be detected about 4 days later in the gut weight matrix. This suggests that some significant change has taken place in person 2’s system at around this time and that the change has influenced both the gut and the tongue microbiomes.

**Fig. 7:**
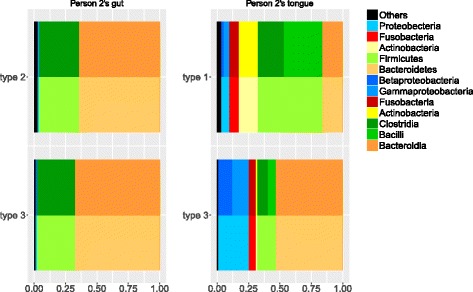
Class and phylum proportions in gut and tongue type matrices. The left panels contain two types from person 2’s gut data and the right panels are for his tongue data. The top plots present the dominant types at the beginning in the time series plot. The bottom plots present the dominant types after the shift in the time series plot. Similar colours in classes are from the same phylum

In order to compare the changes which we have identified as taking place in these microbiomes, the distributions of different phyla and classes of OTUs in each feature are presented in Fig. 7. The top features in this plot are the ones that are more represented in the earlier part of the data (i.e. type 2 for the gut data, and type 1 for the tongue data). The bottom features in this plot are those that are more represented in the later part of the data.

Figure 7 shows a similar shift of composition between the two features for both gut and tongue. In both cases, the type which was more represented in the earlier part of the study has a lower proportion of Bacteroidia and a higher proportion of Clostridia. The proportion of Bacteroidia increases and the proportion of Clostridia decreases for both representative features of gut and tongue data in the later part of the study. The consistency of these changes between the two datasets gives further support to our conjecture that this represents a systematic change at this time. The differences between the types are more pronounced in the tongue data. This could be because the tongue is more exposed to external influences, so its microbiome may be more variable. It might also be because we were using four types to model the tongue data and only three for the gut data. Fitting more types gives the types more room to spread out, allowing for more extreme types and amplifying the differences between the fitted types.

We see that the changes shown in Fig. 7 are consistent with the earlier interpretation of the types in Fig. 5. We used four types here to model the microbiome, but we can see in Fig. 7 that the dominant type after the transition includes much higher abundances of Bacteroidetes (including *Porphyromonas* and *Prevotella*, which has been associated with Periodontal disease [33]) and Proteobacteria (including *Neisseria* and *Haemophilus*) and lower levels of Firmicutes (including *Streptococcus* and *Veillonella*) and Actinobacteria (including *Rothia* and *Actinomyces*). Note that the types in Fig. 5 are fitted from the training data, which is entirely before the state change in person 2.

**Fig. 8:**
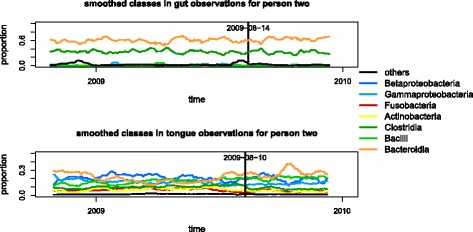
Moving average of class proportions in gut and tongue observations

Having identified the state change using NMF, we ask whether NMF was a necessary tool for identifying the change. First, we compare a naive examination of the composition of the microbiome by class. Figure 8 shows the smoothed proportion of each class over time in person 2’s gut and tongue microbiomes. We see that there are no clear changes in composition at this level, indicating that this is not an obvious change to identify.

For comparison, we also use UniFrac and PCoA for person 2’s gut and tongue data. We see that the first three principal coordinates for the gut data and the first four coordinates for the tongue data do not reveal this change. It is only when we examine the 4th principle coordinate for the gut data and the 5th principle coordinate for the tongue that we are able to detect the changes. The difficulty of finding this explains why this pattern was not found in the many previously published analyses of these data. This is made more difficult by the common practice of examining only the first three principal coordinates. It is possible to find the pattern using UniFrac, if one knows what to look for, but NMF certainly makes the pattern much easier to find.

### The Qin data

The Qin dataset [28] contains human gut metagenome samples extracted from 99 healthy people and 25 IBD patients. The data include 2804 different reactions.

#### Unsupervised NMF results for Qin data

**Fig. 9:**
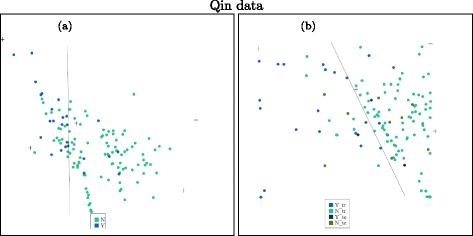
Left: unsupervised NMF based on 6 types. The blue points are from IBD patients and the green ones are from healthy people. Right: supervised NMF on both training and test data. The blue points are training data from patients, and green points are training data from healthy people; the dark blue points are test data from patients, and the dark green points are test data from healthy people. **a** Unsupervised NMF. **b** Supervised NMF

We choose the number of types for the Qin data as six and apply unsupervised NMF on the data. A projection of the data onto a plane is shown on the left panel of Fig. 9. From the plot, we can see that about 19 of the IBD patients can be separated from healthy people. The separation is similar to the results of BiomeNet [15]. The plot shows that two of these features are more related to IBD patients and the other four more related to healthy people. This is consistent with what we find using supervised NMF.

#### Supervised NMF results for Qin data

The sample size of patients is much smaller than the sample size of healthy people. So we perform a classification giving the patients a weight of 4 to balance the class sizes. (For supervised NMF, these weights do not affect the fitted matrices *T* and *W*, only the classifier applied to the weight matrix *W*.) This means that the classifier that assigns all samples to one class will have an accuracy of about 50%. We perform a 10-fold cross-validation on the whole data. Each time, we use nine folds as training data and the remaining observations as test data.

We find two types are enough for patients and four types for healthy people. We perform supervised NMF and fit a logistic regression using the training data weight matrices (with patients given weights of 4) and perform a prediction on the test data. The average of the weighted prediction error over the 10 folds is 0.233 with a standard error of 0.0487.

The projections of both training and test data in one fold of the 10-fold cross-validation are plotted in the right panel of Fig. 9. It shows a quite good separation between these two groups. The classification is not perfect, but is an improvement upon previous methods, such as BiomeNet [15].

The comparisons with support vector machine and random forest methods are summarized in Table 1. The dataset is split to the same 10 folds as supervised NMF. The best parameters are tuned by a 10-fold cross-validation on the whole dataset. The best cost parameter in SVM function is 3 for radial basis kernel and 1 for other three kernels. The best gamma parameter is 10^−4^ for radial basis kernel, 0.1 for polynomial kernel and 0.001 for sigmoid kernel. No method performed significantly better than supervised NMF.

#### Interpretation of the results

**Fig. 10:**
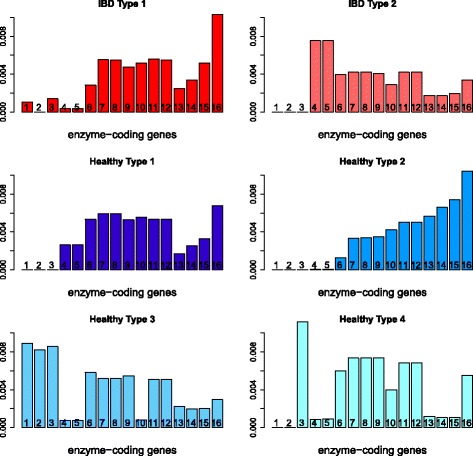
Qin data: the distribution of each type over major enzyme-coding genes: *IBD Type 2* typically represents a group of IBD patients and *Healthy Type 2* represents a group of healthy individuals with these two types distributed very differently over the enzyme-coding genes

**Fig. 11:**
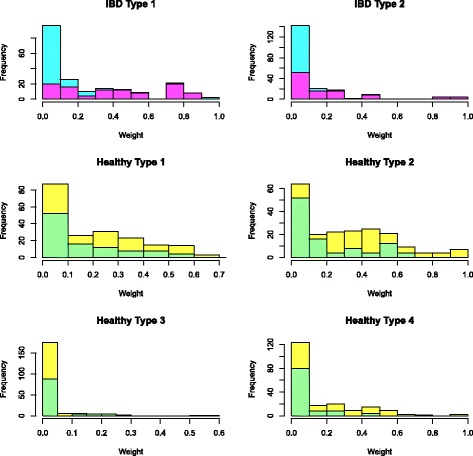
The weights distribution over each type for heathy individuals (top for each bar) and IBD patients (bottom for each bar): the IBD patients mainly have non-zero weights on *IBD Type1*, *IBD Type2*, *Healthy Type 1* and *Healthy Type 2*, and healthy individuals mainly have non-zero weights on *Healthy Type 1*, *Healthy Type 2* and *Healthy Type 4*

The six type vectors are highly sparse with each vector sum equal to 1. We use a cut-off of 0.5*%* for each type to find the distribution of each type over the major reaction groups. Here, each reaction group includes the different reactions that correspond to the same enzyme-coding gene; thus, each category can also be understood as corresponding to one enzyme-coding gene. The type distribution over 17 enzyme-coding genes or reaction groups is shown in Fig. 10. We can observe that the *IBD Type 2* is quite different from other types, with large abundance on the fourth and fifth enzyme-coding genes and that both IBD types have weight zero on the second enzyme-coding gene. Each individual’s metagenome profile is expressed as a linear combination of these six types; the weight distribution over each type is shown in Fig. 11, where the top part of each bar presents the distribution of the weights for healthy individuals for the corresponding type, and the bottom part of each bar is for the weight distribution of IBD patients with each patient counted as four times to make the results comparable to the healthy individuals. From Fig. 11, we can see the IBD patients mainly have non-zero weights on *IBD Type1*, *IBD Type2*, *Healthy Type 1* and *Healthy Type 2*, and healthy individuals mainly have non-zero weights on *Healthy Type 1*, *Healthy Type 2* and *Healthy Type 4*. It seems that the *IBD Type 2* typically represents a group of IBD patients and *Healthy Type 2* represents a group of healthy individuals with these two types distributed very differently over the enzyme-coding genes shown in Fig. 10.

According to Fig. 10, the first three reaction groups contribute more to healthy types and the fourth and fifth reaction groups contribute more to IBD patients (mainly to *IBD Type 2*). Reactions in the first group are all in macrolide biosynthesis. Macrolides are protein synthesis inhibitors and can be used as an antibiotics treatment of inflammatory diseases including inflammatory bowel disease [34–36]. The second reaction group is involved in polycyclic aromatic hydrocarbon degradation and the third group is in carotenoid biosynthesis. Polycyclic aromatic hydrocarbons (PAHs) are one family of ubiquitous environmental toxicants. This family has contributed significantly to the development of colorectal cancer (CRC), a disease highly linked to IBD [37]. Carotenoids can enhance the human immune system’s effectiveness [38]. As IBD is a kind of autoimmune disease, this could explain why these two compounds are lower in IBD patients’ features. The fourth group in *IBD Type 2* is involved in ascorbate and aldarate metabolism and the fifth group in amino sugar and nucleotide sugar metabolism, fructose and mannose metabolism, glycolysis and gluconeogenesis and additional pathways. These are concordant with BiomeNet’s findings in subnetworks 38, 64 and 73. Comparing our reaction groups with the three subnetworks, we notice that reaction group 4 can be found in subnetwork 64 and group 5 has some overlaps with subnetwork 38 and subnetwork 73. These three subnetworks were discovered to have a larger contribution to IBD samples than healthy ones.

### Simulations

#### Simulation based on NMF

We perform simulations in this section to evaluate the performance of our proposed method with regard to the number of types selected and prediction accuracy. We use types estimated from the Qin data [28] to do the simulation. We simulate data according to our proposed model. The data follows a Poisson distribution with mean (*TW*)_*ij*_. To generate these data, we first generate the mean *TW*.

The mean is a linear combination of different features (different columns of *T*). We fix *T* to be the features obtained by applying NMF to the two classes in the Qin dataset [28].

We generate the *W* matrix by generating each entry from a uniform distribution on [ 0,1], then normalizing the column vectors so that the column sums of *W* are equal to the column sums of the IBD data.

The product *TW* gives us the mean, and we add four levels of noise to the product *TW*. The noise is normally distributed with mean 0 and four different standard deviations, to study the effects of different signal-noise ratios (SNR).

$$SNR=+\infty : {sd}_{0}=0 $$

$$SNR=4 : {sd}_{1}={sd}(T)/4 $$

$$SNR=2 : {sd}_{2}={sd}(T)/2 $$

$$SNR=1 : {sd}_{3}={sd}(T) $$

Here, the *sd*(*T*) is a vector of standard deviations for each row of *T*. This is a vector of length p (the number of genes or OTUs) which measures the variability for each gene or OTU across different features in *T*.

The column of *TW* plus the noise is the Poisson mean we use in the simulation. Each element of *X* is generated following an independent Poisson distribution with the mean given by the mean matrix described above.

We simulate data with number of types equal to 2, 5, 10 for class 1 and 3, 6, 9 for class 2. So the number of different combinations is 9 in total. They are 2&3, 2&6, 2&9, 5&3, 5&6, 5&9, 10&3, 10&6, 10&9. Considering the different noise levels, we have 36 scenarios. For each scenario, we simulate 25 replicates. In each replicate, we simulate 200 observations for each class. Then, we separate the data into two parts: the first 200 observations (100 from each class) as the training data and the other 200 as the test data.

We choose the number of types from the training data using a 10-fold cross-validation. After the number of types is chosen, we perform a prediction on the test data using the trained logistic regression model on the training data based on the chosen number of types for each simulated data set.

The NMF, RF and SVM prediction errors are shown in Tables 2, 3 and 4 respectively for different noise levels. We find when the true numbers of types get larger, the NMF prediction errors tend to increase but the RF prediction errors tend to decrease. That may be because we have more accurately estimated the number of types in the cases when the true numbers of types are small. But overall, the prediction errors are quite small for all cases which means our supervised NMF method works well in prediction. NMF performs better in prediction than RF when number of types is small and better than SVM in all scenarios.

**Table 2:** NMF mean prediction test errors for 25 data sets with the standard errors for the mean prediction errors (mean/SE)

SNR	class 1 ∖class 2	2 types	5 types	10 types
+*∞*	3 types	0.0002/0.001	0.004/0.0066	0.0104/0.0126
4		0.0002/0.001	0.005/0.0085	0.0092/0.0102
2		0.0002/0.001	0.0048/0.0071	0.0102/0.0104
1		0.0002/0.001	0.0022/0.0041	0.0084/0.0079
+*∞*	6 types	0.0012/0.0036	0.0054/0.0071	0.0082/0.0089
4		0.0012/0.0030	0.0052/0.0076	0.0154/0.0114
2		0.001/0.0029	0.0056/0.0082	0.0058/0.0067
1		0.0014/0.0037	0.0058/0.0081	0.0126/0.0107
+*∞*	9 types	0.0106/0.0132	0.0068/0.0084	0.0108/0.0126
4		0.0088/0.0102	0.0066/0.0100	0.0132/0.0100
2		0.0078/0.0101	0.0072/0.1011	0.0128/0.011
1		0.0046/0.0058	0.0062/0.0092	0.0124/0.0089

The rows are the true number of types for class 2, and the columns are the true number of types for class 1

**Table 3:** RF mean prediction test errors for 25 data sets with the standard errors for the mean prediction errors (mean/SE)

SNR	class 1 ∖class 2	2 types	5 types	10 types
+*∞*	3 types	0.0024/0.001	0.0012/0.0007	0.0012/0.0005
4		0.0014/0.0004	0.0002/0.0002	0.0012/0.0005
2		0.0016/0.0006	0.0016/0.0006	0.0006/0.0003
1		0.0018/0.0006	0.0008/0.0004	0.001/0.0005
+*∞*	6 types	0.0022/0.0007	0.0026/0.0009	0.0008/0.0005
4		0.002/0.0007	0.0018/0.0008	0.001/0.0006
2		0.002/0.0008	0.0014/0.0005	0.001/0.0005
1		0.002/0.0008	0.0024/0.0008	0.0006/0.0004
+*∞*	9 types	0.0028/0.001	0.0014/0.0007	0.001/0.0006
4		0.0022/0.001	0.0014/0.0006	0.0014/0.0007
2		0.0022/0.0009	0.001/0.0005	0.0008/0.0004
1		0.0022/0.0007	0.0008/0.0004	0.001/0.0005

**Table 4:** SVM mean prediction test errors for 25 data sets with the standard errors for the mean prediction errors (mean/SE)

SNR	class 1 ∖class 2	2 types	5 types	10 types
+*∞*	3 types	0.1462/0.0173	0.13/0.0167	0.1276/0.0156
4		0.1356/0.0176	0.1784/0.0169	0.1616/0.0153
2		0.1356/0.0176	0.1784/0.0168	0.146/0.0124
1		0.1356/0.0176	0.185/0.0151	0.146/0.0125
+*∞*	6 types	0.1794/0.018	0.1214/0.0146	0.162/0.0154
4		0.1774/0.0191	0.1626/0.0154	0.1738/0.0164
2		0.1772/0.0191	0.1498/0.0147	0.159/0.0172
1		0.1392/0.0173	0.1632/0.0154	0.1248/0.0151
+*∞*	9 types	0.1344/0.0193	0.1656/0.0179	0.174/0.0177
4		0.121/0.0158	0.1558/0.0166	0.1846/0.0162
2		0.121/0.0158	0.1552/0.0181	0.1824/0.0177
1		0.121/0.0159	0.1652/0.0198	0.1794/0.0178

Table 5 summarizes the results of the number of types chosen. It shows that the algorithm tends to output slightly larger values than the true number of types in most scenarios, but the true numbers of types mostly are within one standard deviation of the mean of the chosen number of types. Note also, the number of types are chosen only by performing the Wilcoxon Rank-Sum test for each class (see the appendix), the results are not modified through optimizing the classification results based on combined types.

**Table 5:** Simulation summary of the estimated numbers of types

SNR	2			5		10	
		Mean	sd	Mean	sd	Mean	sd
+*∞*	3	2/3.4	0/1.0	5/3.1	0.3/0.3	8.9/3.5	1.9/1.3
4		2/3.8	0/1.5	5/3.3	0.3/1.2	8.9/3.7	2.2/2.0
2		2/3.7	0/1.4	5/3.1	0.3/0.3	8.9/3.7	2.2/2.0
1		2/3.6	0/1.4	5.1/3.0	0.5/0.2	8.5/3.8	1.9/2.0
+*∞*	6	2/6.4	0/0.8	5/6.4	0.5/0.7	9.8/6.6	1.2/1.2
4		2/6.4	0/0.6	5.2/6.6	0.8/0.9	9.5/6.4	1.3/0.6
2		2/6.4	0/0.6	5.2/6.6	0.5/0.8	9.4/6.3	1.5/0.5
1		2/6.3	0/0.6	5.0/6.6	0.5/0.8	9.4/6.2	1.1/0.5
+*∞*	9	2.4/8.4	1.5/1.5	5.1/8.9	1.7/0.8	8.7/9.2	1.8/0.8
4		2/8.2	0/1.7	5/9.8	0.4/1.1	8.8/9.2	1.8/0.6
2		2.4/9.1	1.5/0.7	7.0/9.3	1.5/0.8	11.0/10.9	1.2/1.4
1		2.5/9.0	1.1/1.1	6.9/8.7	1.8/0.9	9.7/10.8	1.7/1.8

For example, the first entry 2/3.4 means when the true number of types is 2 for class 1, 3 for class 2 and *SNR*=+*∞*, the mean numbers of types our method chooses are 2 for class 1 and 3.4 for class 2

Table 5 also shows that in most replicates, when the noise level becomes higher, the difference between the mean and the true number of types will increase. Nevertheless, these results demonstrate that our method is quite effective in finding the appropriate number of types.

Further simulation results (not shown in this paper) have shown that when we apply NMF with the true number of types on the simulated data, the features computed from the data can match very closely with the true features that were used to generate the data. Applying NMF with the wrong number of features can recover a space with the true features embedded in it. The study of consistency of the NMF method is not a trivial topic and deserves further research.

#### Simulation with outliers

We designed this simulation to measure how our method performs when the data contain outliers. We perform this simulation based on data generated in the last section. We use the generated data of scenarios 2&3 types, 5&6 types and 10&9 types, with *SNR*=1. We generate outliers by mislabeling the class of observations in the training data. We run simulations with 5, 10 and 20% of observations in the training data mislabeled. We used the same procedure as in the previous section to calculate the prediction errors. The results in Table 6 show that while RF is more robust in this simulation, NMF still predicts fairly well when there are outliers in the data.

**Table 6:** Mean prediction test errors and the associated standard errors (mean/SE) for simulation with outliers

	Number of types			
Outliers proportion	Method	2&3 types	5&6 types	10&9 types
5%	NMF	0.015/0.0029	0.0396/0.0054	0.0582/0.0063
	RF	0.0042/0.0012	0.0036/0.0009	0.0028/0.0009
	SVM	0.1874/0.0102	0.187/0.0117	0.2114/0.0145
10%	NMF	0.0266/0.0040	0.0564/0.0050	0.089/0.0061
	RF	0.0098/0.0024	0.0094/0.0020	0.008/0.0018
	SVM	0.2404/0.0101	0.2298/0.0120	0.2694/0.0126
20%	NMF	0.075/0.0088	0.1424/0.0083	0.161/0.0084
	RF	0.0338/0.0037	0.0264/0.0042	0.0308/0.0036
	SVM	0.3066/0.0110	0.302/0.0147	0.3118/0.0158

#### Simulation with zero inflated weight matrix

In the previous simulations, we generated the weight matrix of the Poisson mean from the uniform distribution. The sparsity of the generated datasets is around 24%, which is less than is typically observed in practice. We therefore use a Dirichlet distribution with all parameters 0.005 for the weights, in order to generate zero-inflated data. This results in a sparsity of around 39%. We follow the same steps from the first section of the simulations, to generate 36 scenarios and 25 replicates in each scenario. The prediction errors and the associated standard errors of NMF, RF and SVM are shown in Tables 7, 8 and 9. The results show that NMF and SVM are robust when the data become more sparse. RF performs worse in this simulation than in the original simulation.

**Table 7:** NMF mean prediction test errors and the associated standard errors for simulation with zero-inflated weight matrix

SNR	class 1 ∖class 2	2 types	5 types	10 types
+*∞*	3 types	0/0	0.0048/0.0016	0.0158/0.0023
4		0/0	0.005/0.0016	0.0154/0.0023
2		0/0	0.005/0.0016	0.015/0.0024
1		0/0	0.005/0.0016	0.0148/0.0021
+*∞*	6 types	0.0016/0.0009	0.0062/0.0017	0.0148/0.0025
4		0.0016/0.0009	0.0058/0.0076	0.0144/0.0024
2		0.0014/0.0008	0.0066/0.0018	0.0148/0.0024
1		0.0018/0.0010	0.0062/0.0016	0.0144/0.0025
+*∞*	9 types	0.0033/0.0013	0.0068/0.0017	0.0148/0.0024
4		0.011/0.0018	0.0058/0.0015	0.0134/0.0022
2		0.0036/0.0013	0.0066/0.0015	0.0142/0.0026
1		0.0032/0.0011	0.0078/0.0024	0.0156/0.0023

**Table 8:** RF mean prediction test errors and the associated standard errors for simulation with zero-inflated weight matrix

SNR	class 1 ∖class 2	2 types	5 types	10 types
+*∞*	3 types	0.0072/0.0015	0.0062/0.0015	0.0046/0.0012
4		0.0054/0.0013	0.0026/0.0010	0.0042/0.0015
2		0.0048/0.0013	0.0058/0.0012	0.0044/0.0012
1		0.0068/0.0021	0.0056/0.0013	0.005/0.0012
+*∞*	6 types	0.0074/0.0013	0.0122/0.0021	0.0082/0.0018
4		0.009/0.0017	0.0118/0.0014	0.0066/0.0013
2		0.0112/0.0021	0.0148/0.0024	0.0076/0.0018
+*∞*	9 types	0.0102/0.0019	0.015/0.0024	0.0118/0.0023
4		0.011/0.0018	0.0168/0.0024	0.0104/0.0021
2		0.0104/0.0017	0.015/0.0030	0.0122/0.0028
1		0.01/0.0019	0.0126/0.0022	0.014/0.0026

**Table 9:** SVM mean prediction test errors and the associate standard errors for simulation with zero-inflated weight matrix

SNR	class 1 ∖class 2	2 types	5 types	10 types
+*∞*	3 types	0.1224/0.0166	0.2022/0.0193	0.1622/0.0171
4		0.113/0.0150	0.1776/0.0188	0.1884/0.0162
2		0.1324/0.0163	0.169/0.0205	0.1948/0.0144
1		0.1222/0.0134	0.204/0.0158	0.1896/0.0134
+*∞*	6 types	0.1246/0.0146	0.1818/0.0179	0.1596/0.0144
4		0.1254/0.0141	0.2048/0.0112	0.22/0.0125
2		0.1262/0.0149	0.2088/0.0129	0.2366/0.0093
1		0.1206/0.0147	0.2084/0.0120	0.2058/0.0128
+*∞*	9 types	0.1744/0.0127	0.2274/0.0157	0.223/0.0123
4		0.175/0.0096	0.1472/0.0173	0.1794/0.0125
2		0.1858/0.0083	0.213/0.0144	0.1882/0.0096
1		0.207/0.01	0.1664/0.0144	0.1742/0.0137

#### Simulation based on Dynamic Ecology Models

The interpretability of NMF is based on the assumption that the microbial community can be interpreted as a mixture of subcommunities. In this section, we study the question of whether realistic community dynamics can give rise to this assumption. Current knowledge of the community dynamics of the microbiome is woefully inadequate; with a few available suggested models, none of which fit the data very well. In this section, we simulate community dynamics under a Holling type II model [39], given by

1 $$\frac{dM_{i}}{dt}=M_{i}\left(r_{i}(1-c_{i}M_{i})+\sum_{j\ne i}\frac{b_{ij}a_{ij}M_{j}}{1+a_{ij}{T_{H}}_{ij}M_{j}}\right)  $$

Here, for OTU *i*, *r*_*i*_ is the intrinsic growth rate; *c*_*i*_ is the coefficient of negative intraspecific interaction, which is the inverse of the carrying capacity of this OTU in isolation; *a*_*ij*_ is attack rate; *T*_*H*__*ij*_ is handling time; and *b*_*ij*_ is the interaction coefficient between OTUs. When *a*_*ij*_ *T*_*H*__*ij*_ *M*_*j*_ is very small, the 1 term dominates the denominator, so the derivative approximately follows generalised Lotka-Volterra type dynamics for these OTUs; when *a*_*ij*_ *T*_*H*__*ij*_ *M*_*j*_ is large such that it dominates the denominator of the fraction, then the term becomes approximately $\frac {b_{ij}}{{T_{H}}_{ij}}$, and the influence of OTU *j* on OTU *i* is limited by this quantity.

The reason we choose the Holling Type II model, rather than the more commonly used generalised Lotka-Volterra dynamics is that the Holling model seems to have more capacity for overlapping communities to coexist without influencing one-another excessively, because the Holling type II model incorporates a limit on the effect of one OTU on another. This makes intuitive sense when the interaction consists of one OTU providing some metabolite to another OTU. We expect the growth of an OTU to be limited by multiple metabolites, and when one metabolite is used up, increasing the supply of another metabolite would not be expected to have a significant increase on the growth rate. This limit on the effect allows overlapping subcommunities to mix in an approximately linear way. We anticipate that a detailed model based on flux balance equations could be developed which would both model community dynamics more accurately and follow the assumptions behind NMF more closely. However, developing new models for the dynamics of microbial ecology is beyond the scope of this paper.

**Fig. 12:**
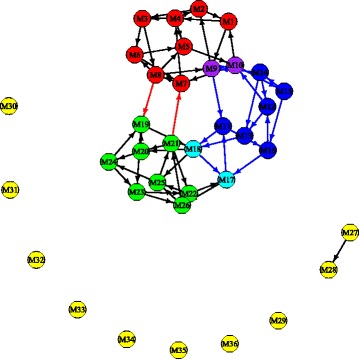
Network used for community dynamics simulations. The red nodes represent OTUs from cluster 1, blue nodes are OTUs from cluster 2, green nodes are from cluster 3 and yellow nodes are isolated OTUs not in any subcommunity. The purple and cyan nodes are overlapping OTUs of cluster 1 and cluster 2, or cluster 2 and cluster 3, respectively

We use the fixed network structure shown in Fig. 12 for the simulations. We can see that the network used is made up from three overlapping clusters (*M*1– *M*10, *M*9– *M*18 and *M*17– *M*26). The intuition is that for each cluster there is a metabolic subcommunity, representing the stable state of the system when restricted to that cluster, and that the overall community is made up as a mixture of these subcommunities. For each black link in the network in Fig. 12, we simulate the species interaction coefficient *b*_*ij*_ as following a uniform distribution between 0 and 0.008. For the blue links in the network, we simulate *b*_*ij*_ from a uniform distribution between −0.002 and 0.008, and for the red ones, we simulate *b*_*ij*_ from a uniform between −0.08 and 0. We set *T*_*H*__*ij*_ around 10^−5^ by generating $\frac {1}{{T_{H}}_{ij}}$ from 10^5^×beta(5,1). This scale of *T*_*H*__*ij*_ allows the Holling type II dynamics to take effect—if *T*_*H*__*ij*_ is much larger, the effect of one OTU on another is limited, so the OTUs become almost independent, losing the subcommunity structure. If *T*_*H*__*ij*_ is much smaller, then the interspecific interaction term is approximately linear, so we get gLV dynamics, which are less suited for overlapping clusters. We allow *r*_*i*_ and *c*_*i*_ to vary between samples in each dataset, with *r*_*i*_ simulated from a uniform distribution between 0 and 1, and $\frac {1}{c_{i}}-1$ simulated from 99×beta(1,2). The idea is that these parameters are related to the suitability of the environment for OTU *i*, so different samples would have different values. The other parameters are kept fixed for all samples, since these represent the inherent ability of these OTUs to interact, so should not be expected to vary greatly between environments. We simulate 10 values of the parameters *b*_*ij*_ for the given network. For each of these simulated values, we simulate one data set with 50 samples, one with 100 samples and one with 200 samples. To construct each sample, we simulate values of *r*_*i*_ and *c*_*i*_ for each OTU and simulate the dynamics from Eq. 1, using 1,000,000 iterations with a stepsize of 0.001.

For each dataset, we apply NMF with four types. We compare the fitted types with the known subcommunities, both visually and using a formal loss function.

We also calculate the co-occurrence networks [40] of the simulated data and compare the results with NMF. The co-occurrence network is produced by calculating the correlation of each pair of nodes in the simulated data. A null distribution for each pair is generated by permuting the abundance of one of the pair and re-calculating the correlation. The resampling is performed 1000 times, and the distribution is used to calculate *p* values. The *p* values are then corrected using Benjamini-Hochberg [41] to control the false discovery rate.

Neither NMF nor co-occurrence networks are designed exactly to identify the network structures or parameters of the Holling model. However, from the network structure in Fig. 12, we see that the network can be reasonably decomposed as containing three large subcommunities (shown in red, blue and green in that figure, with nodes in multiple subcommunities coloured in mixed colours, purple and cyan). Both NMF and co-occurrence networks have some capacity to recover these subcommunities. For NMF, these subcommunities would be recovered as the most abundant OTUs in a type, while for co-occurrence networks, they would arise as connected components in the networks. We can attempt to compare the extent to which the two methods succeed at recovering these subcommunities. This extent is somewhat subjective. For an NMF type, we form clusters of OTUs as the OTUs with abundance above some threshold in that type. For co-occurrence networks, we form clusters as the connected components of the network at a certain significance level. We then choose unions of these clusters to recover the subcommunities used for simulation. To allow comparison, we have defined the following loss function for each true subcommunity to measure how far each such union is from the true subcommunity.

- For each OTU in the subcommunity, but not in the union of clusters, the loss is 1.
- For each OTU in the union of clusters, but not in the subcommunity, the loss is 1.
- For each additional cluster after the first in the union, the loss is 1.

**Fig. 13:**
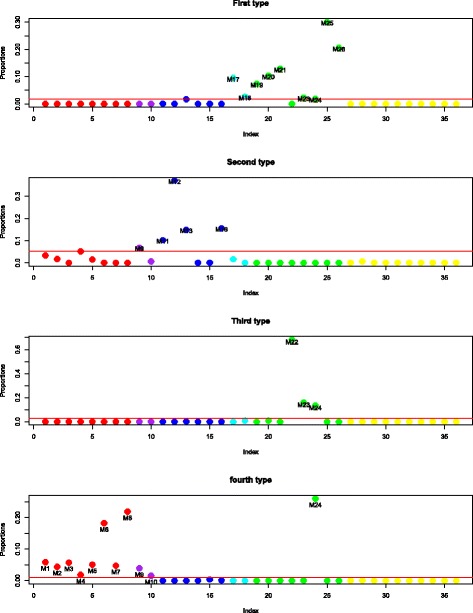
NMF features extracted from data simulated under a Holling type II model

**Fig. 14:**
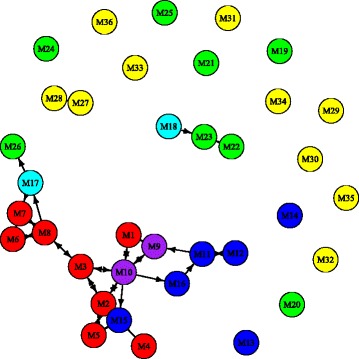
Co-occurrence network calculated from data simulated under a Holling type II model

For example, the loss for the red, green and blue subcommunities in Fig. 13 are respectively 1, 1 and 5, and the loss for the red, green and blue subcommunities in Fig. 14 are respectively 6, 7 and 9 (the blue community being best approximated by a singleton connected component). The abundance thresholds in the NMF type and the significance levels in the co-occurrence networks are chosen to minimise the total loss for each subcommunity. We allow different significance levels for different connected components here. Note that the example calculation above was meant to demonstrate how the loss function is calculated for a given set of clusters, based on the single figure, not on clusters with different *p* values, so the values calculated may not be the actual loss function for that dataset.

Table 10 shows that NMF most often is able to recover the subcommunities used to simulate the data, especially when sample size is large. Note that the blue subcommunity (M9–M18) has weaker interactions between OTUs, so is less clearly a subcommunity, and is therefore not identified as well as the others. Since the loss function is somewhat ad hoc, Figs. 13 and 14 show typical examples of the recovered types from one simulation with 200 data points, to allow more direct comparisons visually. As we can see, NMF has done a better job in recovering the subcommunities. It is also worth noting that co-occurrence networks tend to create many small clusters, which gives the method an advantage over NMF for the above defined loss function, particularly for subcommunities which are not identified well.

**Table 10:** Mean/standard deviation of the optimum loss scores for each true subcommunity under two different methods, with 10 simulations for each sample size

Sample size	Cluster	*M*1−*M*10	*M*9−*M*18	*M*17−*M*26
*n*=50	NMF	3/1.8	7.2/2	2.4/1.9
	Co-occurrence network	5.4/2.4	7.5/0.8	3.5/1.6
*n*=100	NMF	2.7/1.3	6.8/2.2	2.1/1.4
	Co-occurrence network	6.7/2.2	6/1.6	4.5/1.7
*n*=200	NMF	2.7/0.9	6.1/2	1.5/1.2
	Co-occurrence network	6.9/1.7	6.6/2.1	6.3/1.3

The fact that NMF is able to recover the true subcommunities in the simulated data does not necessarily mean that the subcommunities found by NMF on real data are genuine subcommunities, because the dynamics of the real microbial community could be different from those in this simulation. We have however shown that vaguely realistic models of microbial community dynamics can produce subcommunity structures similar to those modeled by NMF. Given that NMF is able to uncover these structures, we have better justification to support that the true community dynamics might also be well represented in terms of the subcommunities identified by NMF and that these subcommunities have meaningful biological structure.

#### Simulation for the performance of NMF as a clustering method

We construct the simulation following the method in McMurdie and Holmes *simulation A* [11]. The real microbial data *ocean* and *feces* from the *GlobalPatterns* dataset are used to obtain two basic sets of multinomial probabilites. We then produce new multinomial probabilites for two classes as linear mixtures of the original two sets. The ratio of these mixtures is determined by the parameter *effect size*, $s_{e}\geqslant {1}$. One class mixes the basic sets in the ratio 1:*s*_*e*_, the other mixes them in the ratio *s*_*e*_:1. When *s*_*e*_=1, the classes are identical, so we expect no separation. As *s*_*e*_ increases, the difference between the classes becomes larger, so the clustering problem becomes easier. We simulate 200 samples for each class with effect size set to 1.01, 1.05, 1.1, 1.3 and 1.5 and sequencing depth set to 10000. For each value of the effect size, we simulate 30 replicates.

For each simulated data set, we calculate the NMF weight matrix on the original count data using two types and then calculate the Euclidean distance between samples based on the NMF weight matrix. For comparison, we also calculate Bray-Curtis dissimilarity, Euclidean distance, weighted UniFrac and rarefied Unweighted UniFrac on proportional data. We perform clustering analysis using Partitioning Around Medoids (PAM) with the number of clusters fixed as two and measure the performance of the methods by the mis-clustering errors. The results are shown in Table 11.

**Table 11:** Mean/standard error of the mis-clustering errors from the 30 replicates

	Effect size				
Method	1.01	1.05	1.1	1.3	1.5
NMF	0.4732/0.0042	0.134/0.0104	0.0003/0.0002	0/0	0/0
Weighted UniFrac	0.469/0.0035	0.0195/0.0012	0/0	0/0	0/0
Unweighted UniFrac	0.4827/0.0034	0.4798/0.0025	0.4887/0.0077	0.4916/0.0082	0.2823/0.0203
Bray-Curtis	0.4683/0.0033	0.2411/0.0128	0.0089/0.0020	0/0	0/0
Euclidean	0.4596/0.0053	0.1607/0.0084	0.0053/0.0010	0/0	0/0

From the table, we see that NMF performs generally better than other methods except weighted UniFrac. This simulation was based on phylogenetically very different classes, which gives UniFrac an advantage, and fixed sequencing depths, which nullifies one of UniFrac’s limitations. Despite this, the results are mostly comparable, and NMF outperforms other commonly used methods. This shows that the dimension reduction by NMF could help to filter out the noise and retain the major dissimilarity signals of the data.

## Conclusion

The NMF analysis can provide a range of interpretable conclusions about the data sets. For metagenomic data, the features extracted can be mapped to metabolic pathways. For OTU data, the features correspond to communities of OTUs and can be studied in terms of the proportion of each phylum, class or genus. In any case, looking at the results of the NMF can reveal important patterns or differences between individuals that are not apparent from the original data. We were able to identify this type of pattern in all three real data sets—the difference in macrolide synthesis pathways for the non-ruminant herbivores; the change in composition of the gut and tongue microbiomes for person 2 in the moving picture data; and the differences in various pathways for the Qin data.

The simulation results show that supervised NMF can recover the right number of types based on which a good classification result can be achieved. Supervised NMF can effectively reduce the dimensionality of the data to a non-negative and most often sparse data matrix, which contains sufficient discriminative information for classification purposes. In addition to the accuracy for classification, these typical features are the community signatures for each class of objects and their interpretation can often uncover important information about the differences between different classes of objects. Simulations of community dynamics under a Holling type II model show that plausible models of community dynamics can lead to the type of additive subcommunity structure assumed by NMF, and that in such a case, NMF is able to identify biologically meaningful types representing the subcommunities.

There are a number of ways the work could be extended in future. The following are some of the most promising and related problems:

Choosing the number of types is still a difficult problem. The method used in this paper can give an answer based on what is needed to make each class different from other classes. However, the non-parametric method has limited efficiency and, as was shown in the simulation, can be quite far from the true values.

NMF fitting does not always have a unique solution. There are a variety of methods in the literature to fix a “best” solution, based on decisions of which aspects of the solution should be penalised. For example, sparsity constraints can be added [42] to make *T* or *W* even more sparse. More work is needed to determine which form of penalty is most appropriate for microbiome data. This penalty could be used to incorporate the phylogenetic structure into NMF. There is a strong intuition in the field that the phylogenetic structure should be important in analysing microbiome data, although there is no clear idea of exactly how it should be used. A penalty could be added to encourage closely related OTUs to be included in the same type. By examining the structure of types for unpenalised NMF, we could gain insight into the appropriate form for this penalty.

As yet, there is no goodness-of-fit test for NMF. That means that we are not certain whether the features identified really represent biologically meaningful entities. There is support for this belief from the fact that they allow us to accurately classify samples and also because the features have a biological interpretation which makes sense. However, a formal test to confirm that NMF fits the model well would be a valuable tool. It would also help with the next topic in our future work.

More theoretical work is needed to justify that NMF can recover the true underlying communities. This is complicated by the non-uniqueness of the solution. Once a method for resolving this non-uniqueness is chosen, it should be possible to identify conditions under which it will recover the true subcommunities, given enough data.

## Appendix A

### Non-negative Poisson regression

Our purpose is to find the non-negative coefficients for a Poisson regression with identity link and without intercept, by maximizing the Poisson log-likelihood. We now focus on the regression of one sample *X*_*j*_=(*X*_1*j*_,*X*_2*j*_,⋯,*X*_*pj*_) on *T*. The resulting coefficients *W*_*j*_=(*w*_1*j*_,⋯,*w*_*kj*_) thus will be either positive or 0, with 0 coefficients corresponding to the variables in *T* removed from this regression. We aim to find a list of positive coefficients with the corresponding variables, so that adding another variable to the list cannot improve the likelihood and still maintain the non-negative constraint. This is achieved through a backwards-forwards Poisson regression procedure as follows.

We start by recursively fitting a Poisson regression on *T* and removing the variables corresponding to the negative coefficients in *W*_*j*_=(*w*_1*j*_,⋯,*w*_*kj*_) until all the coefficients are positive. Using the remaining variables, we calculate the log-likelihood value. Then, we test each removed variable by adding it back with a small positive coefficient, if this increases the log-likelihood value, we add this variable back to the remaining variables and repeat the above steps; otherwise, we remove this variable and test the next one.

The algorithm follows these steps:

1. Fit a Poisson regression with identity link but without intercept on *T* with the initial value of *W*_*j*_ set as the coefficients of linear least square regression of *X*_*j*_ on *T*. Eliminate those variables corresponding to negative coefficients.
2. If any variables were removed, go back to step 1 until all the coefficients are positive. In the end, the matrix consisting of remaining variables is $T^{+}_{j}$. Since *X*, *T* and *W* are all non-negative, the resulting $T^{+}_{j}$ cannot be empty unless *X* is a zero vector.
3. Calculate the log-likelihood for $T^{+}_{j}$.
  $$L\left(T^{+}_{j}\right)=\sum_{i=1}^{p}\left(X_{ij}\log\left(T^{+}_{j}W_{j}\right)_{i}-\left(T^{+}_{j}W_{j}\right)_{i}\right), $$
  where $\left (T^{+}_{j}W_{j}\right)_{i}$ denotes the ith element of the vector $T^{+}_{j}W_{j}$.
4. Add one variable in the removed pool to $T^{+}_{j}$, denote the new feature matrix as ${T^{+}_{j}}_{new}$ and calculate the log-likelihood again.
  $$\begin{aligned} L\left({T^{+}_{j}}_{new}\right)=&\sum_{i=1}^{p}\left(X_{ij}\log\left({T^{+}_{j}}_{new}{W_{j}}_{new}\right)_{i}\right.\\ &\left.-\left({T^{+}_{j}}_{new}{W_{j}}_{new}\right)_{i}\right), \end{aligned} $$
  where *W*_*j*__*new*_=(*W*_*j*_(1−*ε*),*ε*), *ε* is a very small positive number close to 0. For this paper, we use 10^−7^ as the value of *ε*.
5. Compare $L\left (T^{+}_{j}\right)$ with $L\left ({T^{+}_{j}}_{new}\right)$, if $L\left (T^{+}_{j}\right)<L\left ({T^{+}_{j}}_{new}\right)$, use this new ${T^{+}_{j}}_{new}$ composed of $T^{+}_{j}$ and the new variable to repeat steps 1 to 5. Otherwise, remove this variable and try to add another variable in the removed pool to $T^{+}_{j}$ and repeat steps 4 to 5, until all removed variables have been tested.

In step 4, we add back one removed variable each time into the positive T matrix and calculate the new log-likelihood value. To decide if this variable should be added back, we do not need to refit the Poisson regression when calculating the new log-likelihood value. As the old coefficient matrix is a local maximization for the log-likelihood function with the remaining variables, the derivative of the log-likelihood at that point should be 0 with respect to all remaining variables. When we add another variable with a small positive coefficient into the system, if we are near to the original maximum, the log-likelihood for the new point will either increase or decrease, depending whether the derivative with respect to the newly added variable is positive or negative. So if we want to see whether a variable could increase the log-likelihood, we can just add a very small weight *ε* for the new variable, then calculate the new log-likelihood with the new rescaled weight matrix. We need to rescale the *W*_*j*_ vector, so that *Wj*′_*new*_1=*Xj*′1, where 1=(1,⋯,1). This is because we assume the data follow the Poisson distribution, so the sum of the observations *X*_*j*_ should be equal to the sum of the mean vector *TW*_*j*_. As each column of *T* has unit sum, $W^{\prime }_{j}1=W'_{j}T'1=X'_{j}1$.

We compare this new log-likelihood value with the old one. If it decreases, the derivative is negative which means points with positive weight on the new variable will decrease the log-likelihood. Then, the new variable should not be added. If the new one is larger than the old one, add this variable into the positive *T* matrix and do a Poisson regression on this new positive *T* matrix again and repeat the above steps until no variable can be added. In this way, we can make sure that each time we decide to add a new variable to the positive *T* matrix, the likelihood becomes larger. This procedure keeps the log-likelihood function increasing under the constraints that all elements in *W*_*j*_ remain non-negative.

To see that the algorithm will converge, a key point is that our algorithm is only dealing with the discrete part of the optimization, and the Poisson regression takes care of the continuous optimization. Since we are optimizing over a finite number of possible sets of positive variables, convergence is guaranteed by the fact that each step increases the likelihood.

## Appendix B

### Method for choosing the number of types

In order to choose the best number of types for the first class, we will look at the deviance statistics to see how well the chosen types will fit the first class better than other classes. (Deviance is a measure of fit between data and model, given by the difference in log-likelihood between the current model, and a saturated model. Smaller deviance corresponds to better fit.) Since the types are chosen from the first class, to make the comparison objective, the deviance statistics need to be calculated on a test set of the first class. We obtain one deviance statistic for each data point in the test set. We use cross-validation, so that every data point is in one test set. The deviance statistics are not normally distributed; thus, we will use the Wilcoxon Rank-Sum test [43] based on the deviance statistics to test how well the classes are separated. The idea is to rank the deviance statistics from the test data points. If there is no discrimination between the classes, then the ranks should be distributed randomly between the classes. The Wilcoxon Rank-Sum test computes a statistic which measures how unevenly the ranks are distributed between the classes. This statistic is then standardised so that it (approximately) follows a standard normal distribution under the assumption that the ranks are randomly distributed between classes. We refer to this standardised statistic as a *Z*-value. We obtain one *Z*-value for each fold of the cross-validation. Our overall measure of difference is the sum of the *Z*-values for each fold, divided by $\sqrt {r}$, where *r* is the number of folds. (Dividing by $\sqrt {r}$ ensures that if the model is equally good at fitting the data from the two classes, then this overall measure follows a standard normal distribution.) We have one *Z*-value from each fold of the cross-validation, so by calculating the standard deviation of these *Z*-values, we are able to obtain a standard error for our overall statistic. For each class, we will try a sequence of values for the number of types and find the best value to discriminate this class from other classes.

We use a 2-class data case as an example to illustrate the ideas. We use an *r*-fold cross-validation on training data for both classes. In each cross-validation, we separate the training data into a training fold and a test fold. To choose the number of types for class 1, we apply the following steps to a range of values for *k*:

1. For each fixed value *k*, fit *k* types on the training folds from class 1 to get the type matrix *T*.
2. Fit the remaining test fold data from class 1 and one fold of data from class 2 on *T*.
3. Calculate the deviance for each fitting (one deviance value for each data point in the test folds).
4. Use a Wilcoxon Rank-Sum test on these deviances to get one *Z*-value for each fold.
5. Sum the values of *Z* statistics from each fold of the cross-validations and divide by $\sqrt {r}$; denote this statistic as *Z*_*all*_. This statistic should follow a normal distribution with mean of zero and standard deviation of 1 under the null hypothesis that the distributions of deviance values from both classes are the same.
6. Choose the smallest *k* for which *Z*_*all*_ is within one standard deviation of the largest *Z*_*all*_-value, where the standard deviation is calculated as the sample standard deviation of the *Z*-values from the different folds for each *k*.

Note that the purpose is to choose *k* such that the deviances from two classes are best separated, not a hypothesis test to test the equality of means. Thus, the sample standard deviation of *Z*_*all*_ is calculated from the different folds in the last step, instead of using 1, which is the standard deviation under the null hypothesis. By using *r*-fold cross-validation and combined *Z*-values, we can effectively increase the power of this test, which is particularly important when the number of observations is small.

When the classification problem is an easy one, there is a clear separation between the deviances resulting from the class for which we are selecting the number of types and that from other classes. The near complete separation often results in the almost equal *Z*-values from the different folds; thus, the sample standard deviation of *Z*_*all*_ is small. When the classification problem is hard, the resulting *Z*-values from different folds tend to have larger variance. The number of types selected in the easy case usually is small and clear cut; the number of types selected in the harder case usually tends to be large. After we run the above procedure to select numbers of types for all classes, we will fix the number of types for the easy case and select the best matching number of types for the other class so that the misclassification error is minimized.

## Abbreviations

IBD: Irritable bowel disease
NMF: Non-negative matrix factorisation
OTU: Operational taxonomic unit
PCoA: Principal coordinate analysis
PCA: Principal component analysis
